# Attitudes towards the treatment of nausea and vomiting in pregnancy: results from a nationwide online study in Germany

**DOI:** 10.3389/fgwh.2025.1585262

**Published:** 2025-10-31

**Authors:** Mandy Mangler, Kirsten Kuhlmann, Florian Kohlhepp, Theresa Steeb, Wolfgang E. Paulus

**Affiliations:** 1 Klinik für Gynäkologie und Geburtsmedizin, Vivantes Auguste-Viktoria-Klinikum, Berlin, Germany; 2 Frauenarztpraxis Berlin, Berlin, Germany; 3 Medical Affairs, Exeltis Germany GmbH, Ismaning, Germany; 4 Klinik für Frauenheilkunde und Geburtshilfe, Universitätsklinikum Ulm, Ulm, Germany

**Keywords:** nausea and vomiting in pregnancy, cross-sectional study, patient attitudes, information-seeking behavior, pregnancy

## Abstract

**Background:**

Nausea and vomiting in pregnancy (NVP) affect up to 85% of pregnant individuals, predominantly in the first trimester. While most cases are mild, moderate to severe NVP can significantly impair quality of life and require medical intervention. Besides, safety concerns often influence decision-making. This study examines the perceptions, concerns, and information-seeking behaviors of women in Germany regarding NVP and its treatment.

**Methods:**

A nationwide cross-sectional online study was conducted from March 18–28, 2024, targeting pregnant individuals and mothers in Germany via the “*Echte Mamas*” online community. Participants completed an anonymous online questionnaire comprising 15 items covering sociodemographic data, NVP severity (using the PUQE-24 score for pregnant individuals currently suffering from NVP), treatment attitudes, and information-seeking behavior. Data were analyzed using descriptive statistics and subgroup analyses were performed to investigate differences in NVP severity.

**Results:**

Among 506 respondents (completion rate: 83.9%), 81.9% reported experiencing NVP, with 40% reporting moderate, 30.6% mild and 29.4% severe symptoms. Hospitalization was required in 12.4% of cases, predominantly among those with severe NVP, with 76.3% of those receiving medication post-discharge. Participants primarily sought information from physicians (53.4%), the internet (50.6%), and midwives (44.5%), with more severe NVP prompting greater information-seeking behavior. Safety concerns dominated treatment preferences, with participants prioritizing drug approval (on-label prescription) for pregnancy and rapid symptom relief.

**Conclusion:**

This study emphasizes the need for proactive communication from healthcare providers about safe and effective NVP treatments. Tailored, patient-centered strategies that address safety concerns and provide evidence-based guidance are essential for informed decision-making.

## Introduction

Nausea and vomiting in pregnancy (NVP) is a widespread and often challenging condition affecting a significant proportion of pregnant individuals, predominantly during the first trimester. Epidemiological studies indicate that between 50% and 90% of pregnant individuals experience NVP to varying degrees (1–5). In a recent cross-sectional study in a Chinese population prevalence of NVP even exceeded 90% (6). Symptoms of NVP range from mild nausea to more severe vomiting, which can impair daily functioning and lead to complications such as dehydration, weight loss, and electrolyte imbalances. Severe cases of NVP may escalate into hyperemesis gravidarum (HG), a condition that affects approximately 0.3%–3% of pregnant individuals and often necessitates hospitalization due to its severity (7, 8). According to the Windsor consensus, HG is defined by prolonged and severe nausea and vomiting leading to weight loss (>5% pre-pregnancy), dehydration and electrolyte imbalance (9, 10).

The pathophysiology of NVP is not fully understood, but is thought to involve hormonal changes, particularly increases in human chorionic gonadotropin (hCG) and estrogen (7, 8) as well as in GDF15, as has been recently demonstrated (4). These hormonal shifts, combined with individual physiological susceptibilities, lead to the characteristic symptoms of NVP. Diagnostic approaches for NVP include the assessment of symptom severity, often using validated scales like the Pregnancy-Unique Quantification of Emesis (PUQE-24) score, which allows for the categorization of NVP into mild, moderate, or severe forms (11, 12). HG, being the most extreme presentation of NVP, is generally diagnosed based on a combination of clinical symptoms such as intractable vomiting and weight loss exceeding 5% of pre-pregnancy body weight (1, 7, 8).

The management of NVP varies according to severity (1). Mild cases may be managed conservatively through dietary adjustments, lifestyle modifications, and the use of non-pharmacological treatments such as ginger and acupressure. In moderate to severe cases, pharmacological interventions are often required. Common medications used in the treatment of NVP include the combination of pyridoxine (vitamin B6) and doxylamine and other antiemetics like metoclopramide, meclizine or ondansetron (1, 5, 7, 13–16). Despite the availability of effective treatments, there remains a significant concern among pregnant individuals regarding the safety of pharmacological interventions (17). This is particularly true in countries like Germany, where the use of medications during pregnancy is often viewed with caution due to historical events like the Thalidomide incident, which raised long-lasting concerns about drug safety in pregnancy (18).

Understanding patients' attitudes towards NVP is crucial due to its high prevalence and significant impact on quality of life (19, 20). Safety concerns often influence treatment decisions, underscoring the need for patient-centered approaches. Therefore, this study aimed to explore the perceptions, concerns, and information-seeking behaviors of women in Germany regarding NVP and its treatment.

Interestingly, social media platforms provide a unique opportunity to gather real-world insights directly from patient populations (21). This approach enables healthcare providers to better understand patients' experiences, identify unmet needs, and develop communication strategies that address barriers to treatment adoption. Moreover, ongoing dialogues within social media groups can serve as early indicators of emerging trends or concerns. By leveraging social media as a data source, this study seeks to bridge the gap between clinical research and real-world patient experiences. The insights gained will empower physicians to facilitate informed, patient-centered treatment.

## Methods

### Study design

This cross-sectional, nationwide inquiry was conducted between March 18 and 28, 2024. The evaluation aimed to capture a broad spectrum of attitudes toward NVP and its treatment from women across Germany. Participants were recruited through “Echte Mamas”, one of the biggest online communities in Germany for mothers and pregnant individuals. A link to the anonymous online questionnaire was shared on Instagram (@echtemamas) and Facebook (http://www.facebook.com/echtemamas) by “Echte Mamas” to their community.

### Eligibility criteria

Individuals who answered “No” (never pregnant) were automatically screened out and could not proceed with the questionnaire. Only women who were currently pregnant, had been pregnant in the past, or were already mothers were eligible to participate. Additional inclusion criteria were the ability to read and understand German. Participation was voluntary, and all participants provided electronic informed consent before accessing the full questionnaire. Refusals were not documented, and no incentives were offered. Each participant could only take part once in this cross-sectional survey.

### Questionnaire

As no validated inquiry tools for the objective of our study existed, the questionnaire was developed *de novo* based on a literature review and thorough expert consulting of gynaecologists. The study consisted of an online self-administered questionnaire. The questionnaire comprised overall 15 items, divided into 4 sections, namely 1) sociodemographic data, 2) NVP severity, 3) information-seeking behavior, and 4) attitudes towards treatment of NVP. Questions regarding sociodemographic data included age, education level, pregnancy status, and parity. The severity of NVP was evaluated using the Pregnancy-Unique Quantification of Emesis (PUQE-24) score (14, 15). Participants were categorized as having mild, moderate, or severe NVP based on their symptoms in the previous 24 h. Additionally, for those women who were not experiencing NVP in the previous 24 h or mothers who had experienced NVP during their previous pregnancy, retrospective self-assessments were collected. The section on information-seeking behavior assessed where women sought information about NVP treatments (e.g., healthcare professionals, internet, family members, midwives) and what types of treatment information they sought (e.g., conservative treatments, prescription medications). Finally, to investigate treatment attitudes, participants were asked to rank their concerns and preferences regarding pharmacological treatments, including safety, medication approval status, rapid symptom relief, and long-lasting efficacy on a scale from 1 (very important) to 6 (not important). Questions also addressed concerns about tablet size, ease of administration, and number of doses per day. The following questions were ranked according to their importance. Besides this, participants were asked to balance contrary statements on a scale from −100 to 100 regarding safety and quick symptom relief [“The medication is safe to use during pregnancy” (−100) vs. “The medication provides rapid relief” (100)], price and approval of the pharmacological treatment in pregnancy [“The medication is affordable” (−100) vs. “The medication is approved for use during pregnancy” (100)] as well as on-label vs. off-label dosing [“I follow the package instructions strictly” (−100) vs. “I prefer flexible dosing based on symptoms” (100)].

The questionnaire was pre-tested and validated for clarity and comprehension by independent researchers who were not involved in the design of the original questionnaire and a volunteering patient with NVP. Unclear items were thoroughly discussed and rephrased until a consensus on clarity was reached. Based on this feedback, questions were simplified, the questionnaire was shortened and finally, the questionnaire was revised to its final version. The questionnaire can be obtained from the Supplementary Appendix.

### Ethical approval

According to §15 of the “Berufsordnung für Ärzte” (Professional Code of Conduct for Physicians in Germany) and the guidelines of the German Research Foundation (DFG, “Leitlinien zur Sicherung guter wissenschaftlicher Praxis”), purely anonymous, non-interventional online surveys that do not collect identifiable personal data do not require approval by an ethics committee. In line with these regulations, formal ethics approval was not sought.

### Statistical analysis

Following the guidance of Tabachnik and Fidell, we estimated a minimum sample size of *n* = 150 for this exploratory study by multiplying the number of questionnaire items by a factor of 10 (22). To achieve this sample size efficiently, we collaborated with the “Echte Mamas” online community, one of the largest German social media networks for mothers and pregnant individuals, with approximately 600,000 followers on Facebook and 450,000–500,000 followers on Instagram. A preliminary analysis performed by the “Echte Mamas” team indicated that a substantial proportion of their community was actively engaging with content related to pregnancy and breastfeeding, suggesting high relevance of nausea and vomiting in pregnancy (NVP) as a topic. Based on this analysis, it was anticipated that within approximately two weeks, 300–500 eligible participants could be recruited. The survey link was posted once with the contingency plan to repost if fewer than 300 valid responses had been received by the end of the initial two-week period. Within a 2-week period, the Instagram campaign reached roughly 18.000 unique users, while a post on facebook reached 6.600. Ultimately, 603 individuals started the questionnaire, and 506 completed it, exceeding the required sample size. Statistical analyses were conducted with Microsoft Excel 2405 (Microsoft Corporation, 2024) and SPSS (IBM SPSS Statistics version 24, IBM Corporation, Armonk, NY, USA). Data was analyzed using descriptive statistics to summarize sociodemographic characteristics, NVP severity, and treatment preferences. The results were presented as frequencies, percentages, medians, and means with standard deviations, where appropriate. Associations between NVP severity and treatment behaviors were analyzed using chi-square tests. Additionally, for subgroup differences involving more than two groups the Kruskal–Wallis test was applied. A *p*-value of <0.05 was considered statistically significant.

## Results

### Participant characteristics

A total of 603 women participated, with 506 completing the inquiry template, yielding a completion rate of 83.9%. The majority of participants were aged 31–35 years (44.4%, 261/588) and 68% had given birth previously (*n* = 412). Regarding educational background, 36.4% (*n* = 211) of respondents had completed secondary school (*Abitur* or *Fachabitur*), and 28.8% (*n* = 167) held a university degree (Table 1).

**Table 1:** Baseline characteristics of all participants included in the analysis (n = 590). Fourteen respondents who indicated that they had never been pregnant were excluded (n = 14).

Baseline characteristics category	Total *N* = 590	Currently pregnant *N* = 178 Trimester [Median: 22. Weeks of pregnancy (5->40)] 1. 40 (22.6%) 2. 53 (29.9%) 3. 84 (47.5%)	Child <1 year *N* = 212	Child ≥1 year *N* = 200
Frequency of pregnancies until the end of the 1st trimester				
1 time	313 (53.05%)	76	120	118
2 times	203 (34.41)	74	70	59
3 times	58 (9.83%)	21	18	17
≥4 times	14 (2.37%)	5	3	5
Age				
<20 years	4 (0.68%)	0	0	2
21–25 years	30 (5.1%)	11	4	7
26–30 years	163 (27.72%)	52	23	39
31–35 years	261 (43.9%)	80	27	90
36–40 years	107 (18.2%)	29	12	46
>40 years	23 (3.91%)	5	0	15
Highest educational qualification				
Secondary School Certificate *(Hauptschule)*	34 (5.76%)	12	14	8
Intermediate School Certificate *(Realschule)*	166 (28.14%)	50	59	57
High School Diploma *(Abitur)* or University Entrance Qualification *(Fachhochschulreife)*	211 (35.76%)	65	77	69
University Degree	167 (28.31%)	48	57	62
No qualification	2 (0.34%)	1	1	0

NVP, nausea and vomiting in pregnancy; SD, standard deviation.

At the time of data collection, 29.5% (*n* = 178) were pregnant, with 22.6% of these in their first trimester (*n* = 40), 29.9% (*n* = 53) in the second trimester and the majority in the third trimester (*n* = 84, 47.5%). The median gestational age among pregnant participants was 22 weeks, with a range from 5 to over 40 weeks (Table 1).

### NVP prevalence and severity

**Figure 1:**
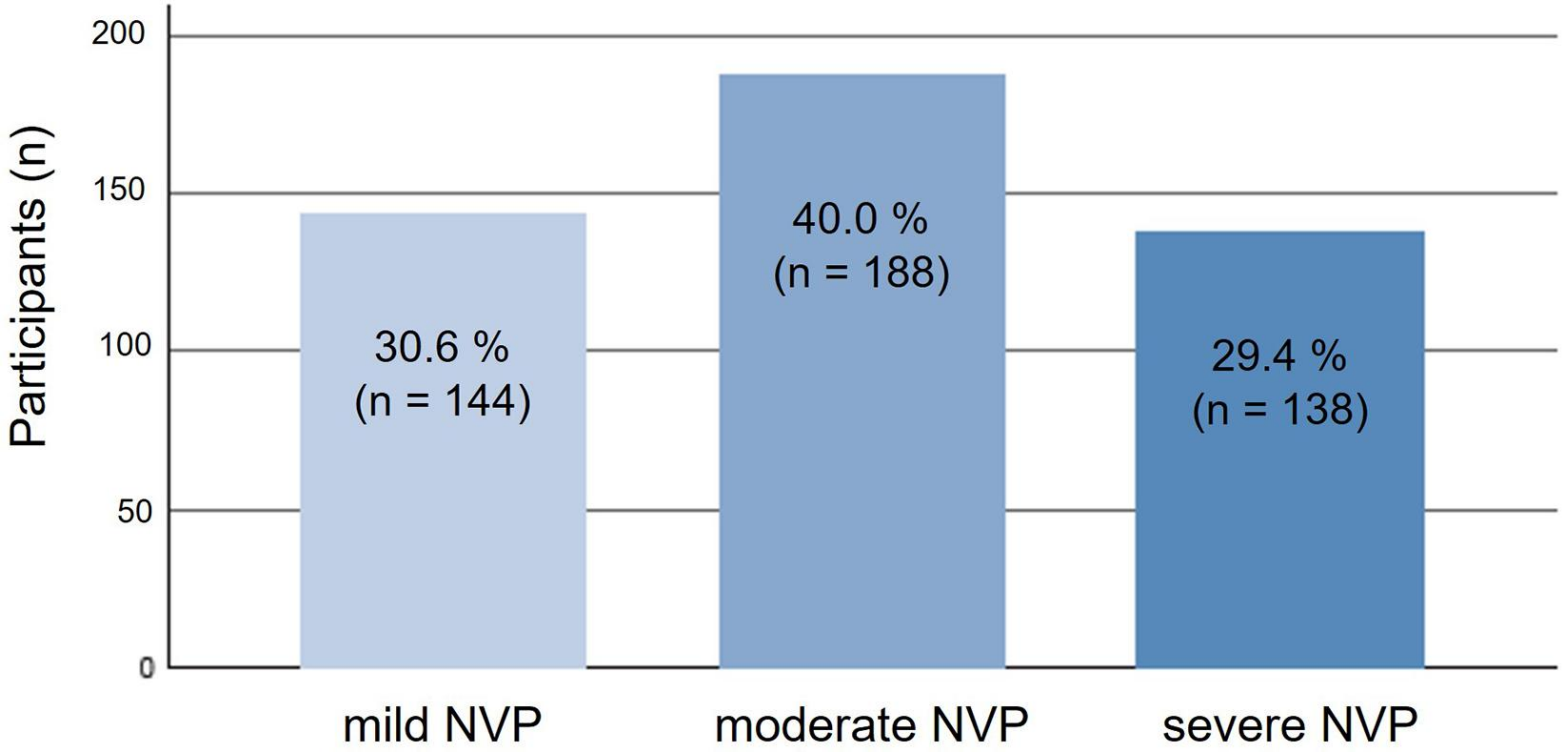
Distribution of participants across severity levels of nausea and vomiting in pregnancy (NVP) as assessed by the Pregnancy-Unique Quantification of Emesis (PUQE-24) score (*n* = 470). NVP, nausea and vomiting in pregnancy; PUQE-24, Pregnancy-Unique Quantification of Emesis score for the past 24 h.

A large majority of pregnant individuals as well as mothers (81.9%, *n* = 476/582) reported experiencing NVP during the first trimester. The severity of NVP varied, with 40% (*n* = 188) experiencing moderate symptoms, 30.6% (*n* = 144) mild symptoms, and 29.4% (*n* = 138) severe symptoms (Figure 1). Among currently pregnant individuals with recent (last 24 h) NVP, the mean PUQE-24 score (rounded) was 8.0 (SD ± 2.55; *n* = 42), indicating moderate severity.

When asked if they had received a prescription to treat their NVP, 273 (58.2%) responded that they had not while 196 (41.8%) voted to have received a prescription. Of those who have received a prescription, 105 (53.6%) with severe NVP indicated to have been treated pharmacologically. Notably, participants with moderate or severe NVP were significantly more likely to receive pharmacological treatment compared to those with mild NVP (*p* < 0.001) (Table 2). Hospitalization due to NVP was necessary for 12.4% (*n* = 59), and 76.3% (*n* = 45) of these received prescriptions for ongoing medical treatment. Notably, participants with severe NVP were more likely to be hospitalized (*p* < 0.001).

**Table 2:** Proportion of participants with nausea and vomiting in pregnancy (NVP) who required pharmacological treatment, stratified by severity based on the Pregnancy-Unique Quantification of Emesis (PUQE-24) score. Percentages refer to the total number of participants (n = 469).

Pharmacological treatment (y/n)		PUQE-24 score			Total
		Mild NVP	Moderat NVP	Severe NVP	
Pharmacological treatment of NVP	Yes	13 (2.77%)	78 (16.63%)	105 (22.39%)	196 (41.79%)
	No	130 (27.72%)	110 (23.45%)	33 (7.04%)	273 (58.21%)

NVP, nausea and vomiting in pregnancy; PUQE-24, Pregnancy-Unique Quantification of Emesis; SD, standard deviation.

### Information-seeking behavior

**Figure 2:**
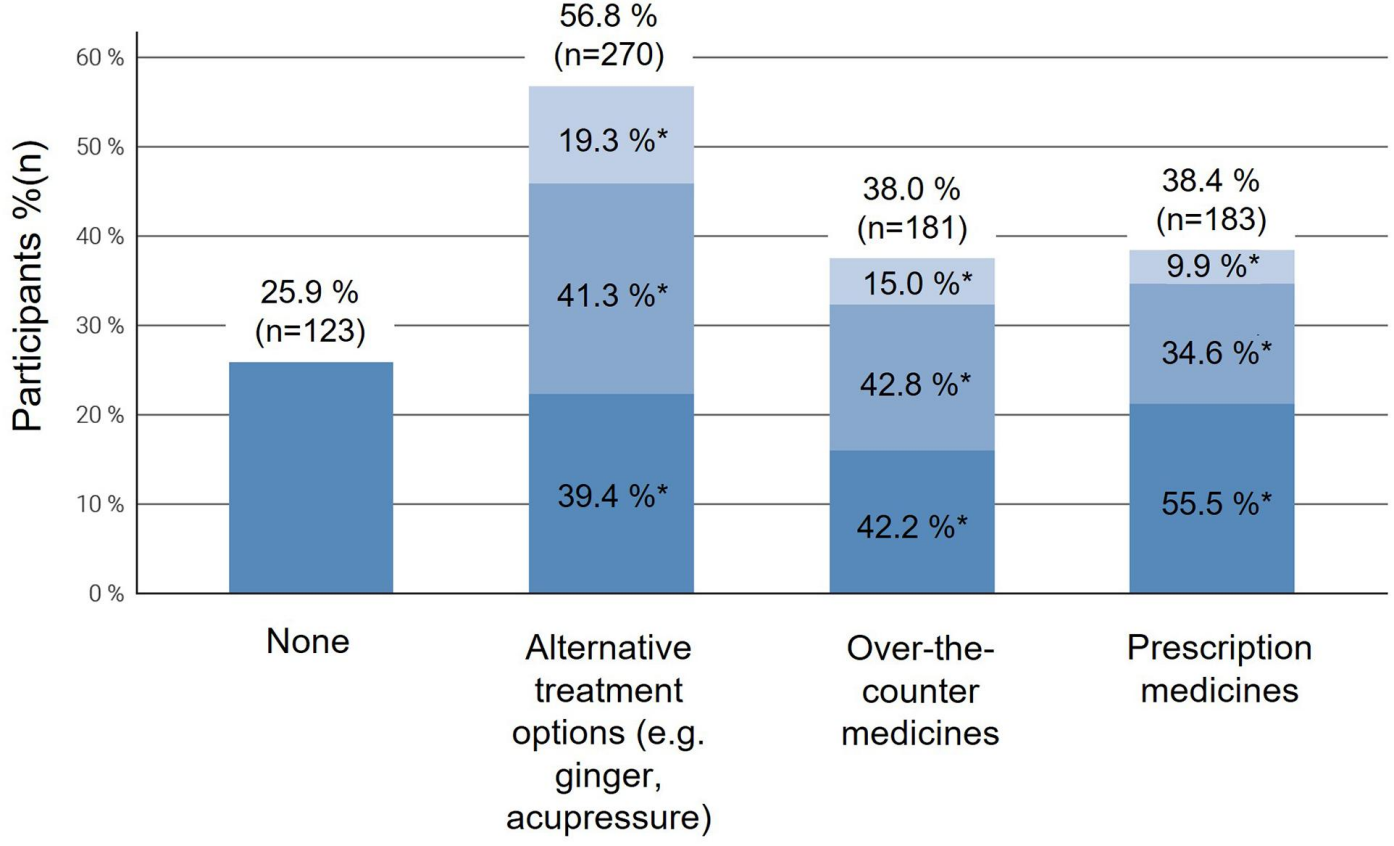
Proportion of participants reporting searching information about the use of different treatment options for nausea and vomiting in pregnancy (NVP), stratified by symptom severity (*mild, moderate, severe*) according to the Pregnancy-Unique Quantification of Emesis (PUQE-24) score. Each column represents the total number of participants using a given treatment type, subdivided by NVP severity: light blue = mild NVP, medium blue = moderate NVP, and dark blue = severe NVP. Multiple responses were possible. NVP, nausea and vomiting in pregnancy.

Overall, when asked about the information-seeking behavior, participants stated to be interested in both conservative treatment options (56.8%, *n* = 270) and prescription medications (38.4%, *n* = 183) as well as over-the-counter medication (38%, *n* = 181). The need for information increased with NVP severity, with participants with moderate to severe symptoms being more proactive in seeking information (*p* < 0.001) (Figure 2). Besides this, participants with a High School Diploma (*Abitur*) or University Entrance Qualification (*Fachhochschulreife*) and those with a university degree were more likely to inform themselves about alternative treatment regimens like ginger or acupressure compared to those with a lower level of education (*p* = 0.003).

**Figure 3:**
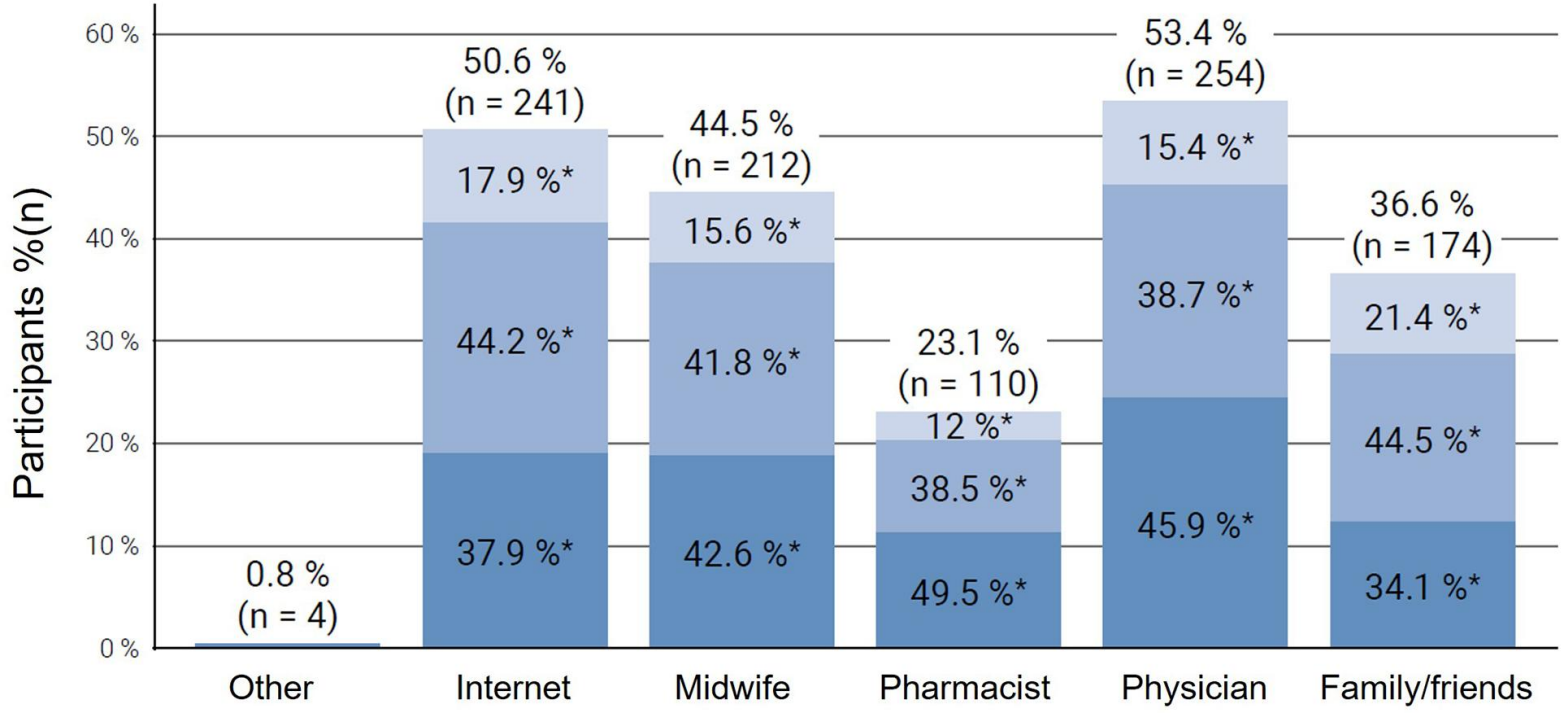
Sources of information consulted by participants regarding treatment options for nausea and vomiting in pregnancy (NVP), stratified by symptom severity (*mild, moderate, severe*) according to the Pregnancy-Unique Quantification of Emesis (PUQE-24) score. Each column represents the proportion of participants using a specific information source, subdivided by NVP severity: light blue = mild NVP, medium blue = moderate NVP, and dark blue = severe NVP. Multiple responses were possible. NVP, nausea and vomiting in pregnancy.

Furthermore, when asked about the most utilized sources of information for NVP treatment, most participants answered to seek information by asking physicians (53.4%, *n* = 254), the internet (50.6%, *n* = 241), and midwives (44.5%, *n* = 212), whereas family and friends (36.6%, *n* = 174) and pharmacists (23.1%, *n* = 110) were consulted less frequently (Figure 3). Further answers obtained from a free text field included asking a Doula (*n* = 1), alternative practitioner (*n* = 1) or consulting the webpage “Embryotox”, where the Pharmacovigilance and Advisory Center for Embryonal Toxicology at Charité-Universitätsmedizin Berlin provides independent information on the tolerability of medicines during pregnancy and breastfeeding. The subgroup analysis revealed that participants with moderate or severe NVP had a greater likelihood of seeking information from family members or friends, physicians, pharmacists, midwives, and the internet than those with mild symptoms (*p* < 0.001). Additionally, the internet was frequently cited by women with mild NVP as a primary source of information.

### Treatment attitudes

When asked about the importance of different treatment attitudes on a scale from 1 (very important) to 6 (not important), rapid symptom relief (mean 2.23 ± 1.35) and long-lasting efficacy (2.62 ± 1.29) were the most important criteria for the pharmacological treatment for women who have experienced NVP (Table 3). The number (4.17 ± 1.42) and size of tablets (4.85 ± 1.51) were less important. Besides this, a simple dosing regimen for the medication (3.53 ± 1.35) and adherence to the dosage regimen according to the patient leaflet (3.01 ± 1.56) were considered to be neither important nor irrelevant. Interestingly, participants with more severe NVP were more likely to prioritize fast-acting medications (2.15 ± 1.26; *p* < 0.001). Thus, the main priorities for pharmacological treatment were quick relief of symptoms and long-lasting effects, with higher importance assigned by participants with severe NVP (Table 3). The subgroup analysis found a statistically significant difference between NVP severity in relation to the size of the tablet (*p* = 0.20) and also to the simple dosing regimen (*p* = 0.21), i.e., the more severe NVP was, the more relevant were those topics for the women.

**Table 3:** Participants' evaluation of the relative priority of various factors influencing pharmacological therapy according to NVP severity. Green shading indicates higher priority, red indicates lower priority.

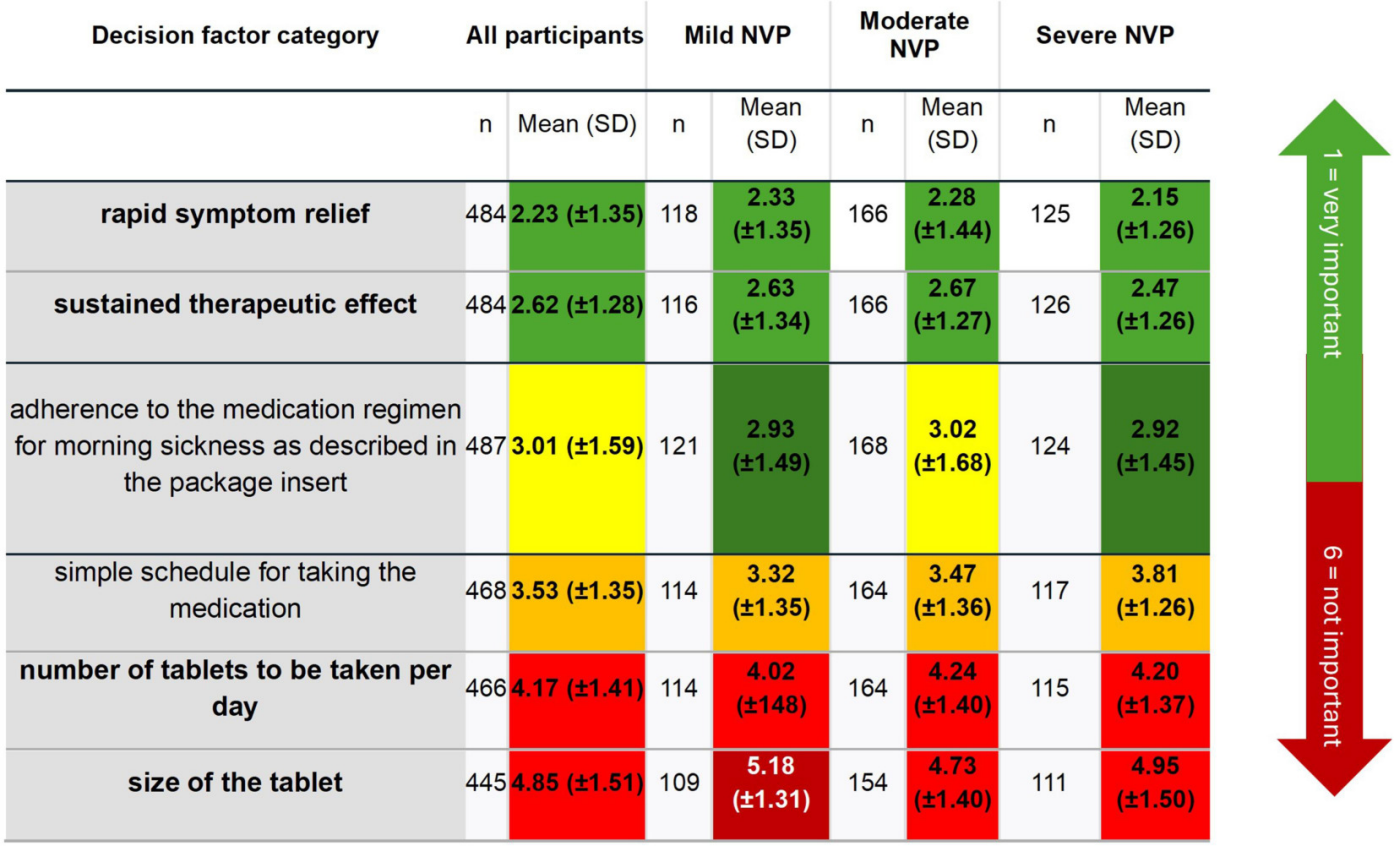

NVP, nausea and vomiting in pregnancy; PUQE-24, Pregnancy-Unique Quantification of Emesis.

Additionally, in the trade-off questions, safety was the most important factor in choosing NVP treatment, with the majority of women who already experienced NVP during the first trimesters expressing concerns about the safety of medications during pregnancy (mean −65.44) and prioritizing it in contrast to rapid symptom relief. Furthermore, drug approval (by regulatory bodies) for use in pregnancy was preferred over cost considerations (mean 87.8). The participants were neutral (mean: 2.76) regarding the statements of strictly adhering to the instructions in the package leaflet and adjusting their treatment based on symptoms and minimizing medication use. Results were similar when including the total population in the assessment and favored safety and approval of the drug in pregnancy (−66.19 vs. 88.0). Regarding dosage of the pharmacological treatment, participants were ambivalent as well (mean 1.62). (Scoring interpretation: 0 = equal importance; negative = preference for safety; positive = preference for the opposing statement.)

## Discussion

This study provides valuable insights into the attitudes and behaviors of women in Germany on NVP focusing on symptom prevalence, treatment-seeking behaviors, and safety perceptions. The observed high prevalence of NVP, particularly during the first trimester (81.9%), is consistent with previous epidemiological studies, which report that up to 85% of pregnant individuals globally experience NVP in early pregnancy (19). This substantial prevalence highlights the need for targeted healthcare strategies that address NVP's impact on maternal health and quality of life. This has already been discussed in studies from other high-income countries where NVP is similarly prevalent (23). Our findings on the prevalence and severity of nausea and vomiting in pregnancy (NVP) are broadly consistent with previous reports but reveal some noteworthy divergences. In large population-based cohorts, NVP is reported by 50%–90% of pregnant individuals (4), with most experiencing mild to moderate symptoms and only 0.3%–3.0% meeting criteria for hyperemesis gravidarum (HG) (3). Recent Asian data show particularly high prevalence rates: Zou et al. reported NVP in 96% of Chinese women, with 7% severe cases by PUQE-24 and up to 27% “significant” symptoms using RINVR (6). This discrepancy may reflect cultural or methodological differences, including online recruitment via the *Echte Mamas* community vs. hospital-based sampling. A recent study from the Czech Republic has also found that lower levels of nausea and vomiting were reported by women who used COC when they met their partner, as well as in those who smoked before pregnancy (24). These differences underline the importance of considering recruitment setting, severity definitions, and population context when interpreting NVP prevalence and its clinical implications.

The distribution of NVP severity in our sample provides a further dimension to understanding the burden of NVP. Our findings indicate that moderate NVP was the most common severity level, affecting 40% of respondents. This aligns with research by Lacasse et al., which reports that while mild symptoms are frequently encountered, moderate and severe forms are highly disruptive and likely to drive healthcare visits and requests for treatment (6, 25). Thus, our study supports the view that moderate symptoms represent a significant burden, indicating that NVP is not just a transient and mild inconvenience but a condition that can interfere substantially with daily activities. Additionally, severe NVP affected 29.4% of participants, a finding consistent with literature indicating that severe cases, though less common, demand more intensive management (6, 26). This highlights the importance of differentiating levels of NVP severity to develop and offer tailored treatment options suited to varying levels of symptom burden (11, 12).

An unexpected finding in our study was the relatively high rate of hospitalization (12.4%) among respondents with NVP. We hypothesized this outcome as a surrogate marker for HG, as this condition is usually treated in the hospital in contrast to NVP. This rate is notably higher than expected for HG, which typically affects less than 0.3%–3% of pregnancies worldwide (7, 8). The discrepancy between our study's findings and typical HG prevalence rates might reflect a lack of standardization in distinguishing severe NVP from HG, an issue also highlighted in the German healthcare system, which lacks comprehensive guidelines for managing NVP at varying severity levels (27, 28). Moreover, this higher hospitalization rate may indicate a need for more robust outpatient care or early intervention strategies, as hospital admissions are likely driven by insufficient symptom control or patient concerns about health risks associated with unmanaged NVP. Previous studies from the US have called attention to this gap in outpatient NVP support, suggesting that proactive management could reduce hospital admissions and improve maternal outcomes (29). Another possible explanation is that women with NVP sought hospital care for their symptoms because they occurred during weekends or at night when physicians were unavailable. Similarly, a shortage of physicians in their area, particularly in rural regions, might have contributed to this phenomenon.

Our analysis of information-seeking behaviors among participants reveals that most women with NVP sought information from multiple sources. Healthcare providers, the internet, and midwives were amongst the most common. This aligns with previous findings, which show that healthcare providers remain primary information sources, yet pregnant individuals increasingly search the internet for supplementary information (30, 31). The internet's prominence reflects a broader trend in digital health information-seeking and underscores the need for accurate online resources. Notably, participants with moderate to severe symptoms showed higher levels of information-seeking. This suggests that symptom severity might correlate with an increased demand for guidance and support. The reliance on online information sources presents both opportunities and challenges. On the one hand, access to digital resources allows for rapid information dissemination and may empower patients. However, it also raises concerns about the quality and accuracy of information, as patients may encounter unverified or anecdotal guidance on unregulated platforms. Given this, healthcare providers play a crucial role in directing patients towards reliable online resources to ensure that they receive credible and evidence-based information.

Participants' attitudes towards pharmacological treatments for NVP highlight a cautious approach, with safety prioritized over rapid relief and medication cost. In countries like Germany, caution regarding the use of medications during pregnancy may stem from historical events such as the Contergan scandal, where the drug Thalidomide caused severe congenital malformations in thousands of children (18). Nevertheless, these findings align with established literature on pregnancy-related medication hesitancy. Evidence suggests that pregnant individuals frequently avoid pharmacological interventions due to concerns over fetal health risks, even when safe treatments are available (23, 32, 33). This has also been shown in a study where 86% of pregnant individuals called a hotline for information on management of NVP with/without questions about fetal drug safety (33). Another study identified reasons for avoiding medication, such as a lack of sufficient safety data, a preference for non-pharmacologic approaches, and discomfort caused by the physician's attitude. Among women who did choose to use medication, the most reassuring and convincing information about its safety came from friends and family (32). Although medications with a proven safety profile for NVP are available, the widespread concern surrounding pharmacological treatments highlights the need for greater transparency and reassurance from healthcare providers. Various studies have shown that safety concerns are often exacerbated by limited awareness of approved medications and their safety during pregnancy (26, 32, 33). Thus, our findings highlight the importance of thorough, evidence-based counseling by healthcare providers addressing common safety concerns and emphasizing regulatory approvals for pregnant individuals.

Interestingly, our findings indicate that participants valued quick and long-lasting symptom relief in NVP treatments, which points to a preference for treatments that effectively manage symptoms with minimal dosing frequency. This is consistent with research indicating that patients prefer treatments that provide sustained relief, particularly for conditions that impact daily functioning (25, 34). However, despite these preferences, the cautious approach to medication uptake remains a barrier to effective symptom management for many patients. Here, individualized counseling that carefully addresses both efficacy and safety concerns might be effective in encouraging appropriate pharmacological use when indicated (29). This dual focus on efficacy and safety could help balance patient concerns with the need for adequate symptom control. This is particularly relevant for those with moderate to severe NVP who may benefit most from pharmacological support.

## Limitations

Although this study offers valuable insights, there are several limitations that should be considered when interpreting the results.

Recruitment exclusively via the social media platform may introduce selection bias towards digitally engaged, higher-education participants, limiting population representativeness.

For currently pregnant individuals who had NVP in the previous 24 h, the PUQE-24 score was determined. The intensity of NVP in currently pregnant individuals without NVP in the previous 24 h and pregnant individuals with a child (<1 year and >1 year) was determined by self-assessment (mild, moderate or severe NVP) and may therefore be susceptible to recall bias. This phenomenon has also been described previously by Koren et al., who showed that women reported significantly more severe NVP symptoms during their follow-up call than they had reported originally (28). Thus, they conclude that retrospective evaluations of NVP symptoms may produce a recall bias, which may distort the evaluation of the therapeutic effectiveness of antiemetics. In addition to the recall bias, retrospective self-ratings are not directly comparable to the standardized PUQE-24 scores, which might affect severity classification.

Furthermore, as the survey was only distributed via the social media platform of the “Echte Mamas” community, i.e., via Facebook and Instagram, selection bias is likely. Thus, the results might not be applicable to a general population of other mothers and pregnant individuals and must therefore be interpreted cautiously. However, the strengths of the study include a large sample size gathered in a very short period, as well as the advantages of utilizing an online community, which provides easy access to a diverse group of participants.

## Conclusion

In conclusion, our findings highlight the importance of personalized, patient-centered management strategies for NVP, particularly for women experiencing moderate to severe symptoms, while recognizing that even mild symptoms can impact daily life and well-being. Proactive communication from healthcare providers is essential in addressing safety concerns and providing evidence-based information. Healthcare providers should be advised to actively engage in counseling to build trust, alleviate fears, and enable informed decision-making.

## Data Availability

The raw data supporting the conclusions of this article will be made available by the authors, without undue reservation.
