# Supplementary material for: Attitudes towards the treatment of nausea and vomiting in pregnancy: results from a nationwide online study in Germany
Source: Front Glob Womens Health. 2025 Oct 31;6:1585262. doi: 10.3389/fgwh.2025.1585262 (PMC12615491; doi:10.3389/fgwh.2025.1585262)
Supplement: Supplementary file 1 [file Table1.docx]

**Questionnaire** **on the topic of Nausea and Vomiting in Pregnancy**

**Question 1:** Are you currently pregnant or have you been pregnant before?
□ Currently pregnant (Follow-up question: Please indicate the week of pregnancy: ___)
□ Child <1 year
□ Child >1 year
□ No (Terminate questionnaire)

**Question 2:** In which week of pregnancy are you?
Specify the week: ___

**Question 3:** How old are you?
Answer in age groups:
□ <20 years
□ 21-25 years
□ 26-30 years
□ 31-35 years
□ 36-40 years
□ >40 years

**Question 4:** How many times have you been pregnant until the end of the first trimester?
□ Once □ Twice □ Three times □ ≥ Four times

**Question 5:** What is your highest educational qualification?
□ Secondary school leaving certificate
□ Intermediate school leaving certificate
□ High school diploma or equivalent
□ University degree
□ No qualification

**Question 6:** Have you ever experienced nausea (and/or vomiting) during the first trimester of pregnancy?
□ Yes (Follow-up questions below)
□ No (If no, answer only general questions: Questions 14, 15)

**Follow-up Question 7:** Were you hospitalized due to pregnancy-related nausea?
□ Yes (Further follow-up: Did you receive a prescription for home treatment? □ Yes □ No)
□ No

**Option 1 (Question 8): If you are currently in the first trimester:**

**PUQE Questions:**
**8A:** How many hours have you felt nauseous in the last 24 hours?
**8B:** How many times have you vomited in the last 24 hours?
**8C:** How many times have you experienced retching in the last 24 hours?

□ Never □ None □ Never
□ 1 hour or less □ 1-2 times □ 1-2 times
□ 2-3 hours □ 3-4 times □ 3-4 times
□ 4-6 hours □ 5-6 times □ 5-6 times
□ >6 hours □ 7 times or more □ 7 times or more

**Option 2 (Question 9): If currently pregnant but no NVP in the last 24 hours:**

How severe were your symptoms during (one of) your previous pregnancy(-ies)?
□ Mild nausea or vomiting (e.g., 2-3h nausea, no vomiting, no retching)
□ Moderate nausea or vomiting (e.g., 4-6h nausea, 1-2 times vomiting, retching)
□ Severe nausea or vomiting (e.g., >6h nausea, 5-6 times vomiting, 5-6 times retching)

**Option 3 (Question 10): If not currently pregnant:**

How severe were your symptoms during (one of) your previous pregnancy(-ies)?
□ Mild nausea or vomiting (e.g., 2-3h nausea, no vomiting, no retching)
□ Moderate nausea or vomiting (e.g., 4-6h nausea, 1-2 times vomiting, retching)
□ Severe nausea or vomiting (e.g., >6h nausea, 5-6 times vomiting, 5-6 times retching)

**Question 11:** Did you treat your pregnancy nausea with doctor-prescribed medications?
□ Yes □ No

**Question 12:** Did you seek information on treatment options for nausea or vomiting during pregnancy? (Multiple answers possible)
□ Yes, on prescription medications
□ Yes, on over-the-counter medications
□ Yes, on alternative treatments (e.g., ginger, acupressure)
□ No

**Follow-up Question 13:** If yes: What sources of information did you use? (Multiple answers possible)
□ Family/Friends
□ Doctor
□ Pharmacist
□ Midwife
□ Internet
□ Others: Please specify _______

**Ranking Question (Question 14):**

How important do you consider the following statements regarding medicinal treatment for nausea and vomiting in pregnancy? Rank from 1 = very important to 6 = not important at all.

1. I follow the dosing instructions on the package insert.
2. Quick symptom relief is important to me.
3. Long-lasting effect is important to me.
4. The number of tablets to take daily is crucial.
5. Tablet size is important to me.
6. The medication should have a simple dosing regimen.

**Comparison Question with Slider (Question 15):**
How do you assess the following statements regarding medicinal treatment for pregnancy nausea? Rank from -100 to 100:

1. The medication is safe to use during pregnancy (-100) vs. The medication acts quickly (100).
2. The medication is affordable (-100) vs. The medication is approved for use during pregnancy (100).
3. I follow the package instructions strictly (-100) vs. I prefer flexible dosing based on symptoms (100).

Thank you for your participation!

[END]
